# Nuclear Pleomorphism in Gastrointestinal Stromal Tumors: Insights Beyond Risk Stratification

**DOI:** 10.7759/cureus.93112

**Published:** 2025-09-24

**Authors:** Subramaniam Ramkumar

**Affiliations:** 1 Pathology, Nazareth Hospital, Shillong, IND

**Keywords:** epithelioid features, gastrointestinal stromal tumor (gist), histomorphology, mitotic index, nuclear pleomorphism, pdgfra, pleomorphic gist, prognostic factors, sdh, spindle cell morphology

## Abstract

Gastrointestinal stromal tumors (GISTs) are very common mesenchymal neoplasms involving the gastrointestinal tract. Despite having a uniform nuclear morphology, they can show varied histological subtypes. The prognosis is based on tumor size, mitotic rate and location of the tumor. Some cases of GIST can however show marked nuclear pleomorphism. Here we report a case of GIST with marked nuclear pleomorphism without other high-grade features. This case highlights the importance of nuclear pleomorphism and its implications in the perspective of existing prognostic criteria.

## Introduction

Gastrointestinal stromal tumors (GISTs) exhibit varied histomorphology, including spindle cell, epithelioid, and mixed histological features [1]. Despite this heterogeneity, nuclear morphology is typically consistent [1]. Prognostic indicators for GISTs include the anatomical site, tumor size, and mitotic index [1]. However, a rare subset of GISTs can exhibit significant nuclear pleomorphism, particularly in nuclear size and shape [2]. This particular tumor variable seen in GISTs can act as a confounding factor and pose as a diagnostic challenge. This morphological deviation highlights the spectrum of cytological diversity that GISTs can occasionally demonstrate, expanding the known histopathological variability of these tumors. In a situation like this, careful interpretation with supportive molecular studies and immunohistochemistry is mandatory to avoid overestimation of tumor grade. Documentation of rare variants such as pleomorphic GISTs is important as it contributes to a better understanding of their prognosis and guides evolving refinements in stratification. In this report, we present a case of GIST with pronounced nuclear pleomorphism and discuss its unique histomorphological features and clinicopathological prognostic implications.

## Case presentation

**Figure 1:**
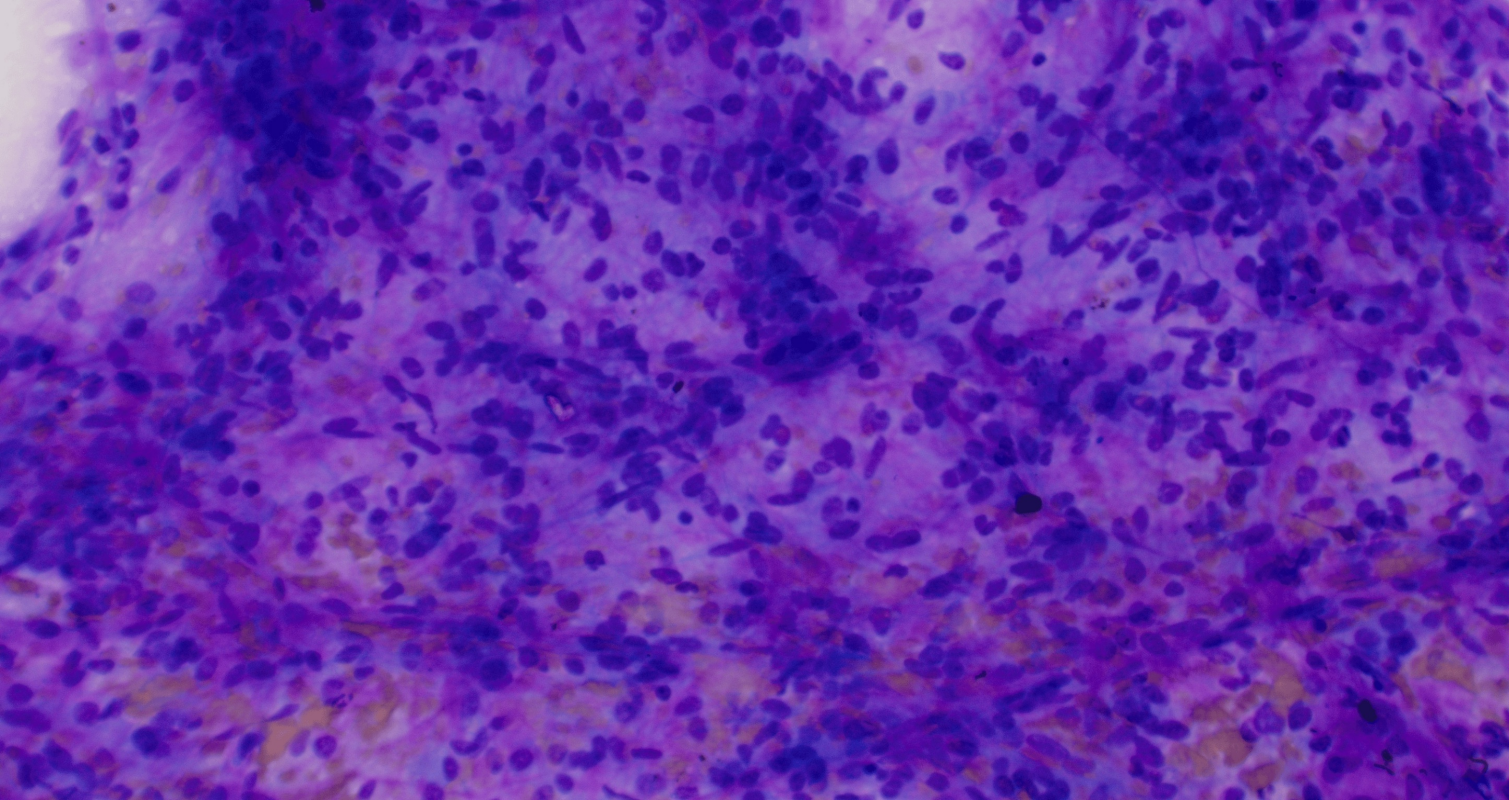
:FNAC showing mixture of spindle and epithelioid cells with minimal mitotic activity (Giemsa x20). No nuclear atypia or necrosis.

A 68-year-old woman with a history of bilateral microinvasive breast carcinoma (pT1), hemochromatosis, hypothyroidism, and sleep apnea was evaluated after computed tomography revealed a heterogeneously enhanced, lobulated mass with smooth borders, measuring 7.8 × 5.3 × 6.7 cm. The mass was located in the middle to upper abdomen near the mesentery. Ultrasound-guided fine-needle aspiration revealed a mix of spindle and epithelioid cells with minimal mitotic activity and no significant nuclear atypia or necrosis (Figure 1).

**Figure 2:**
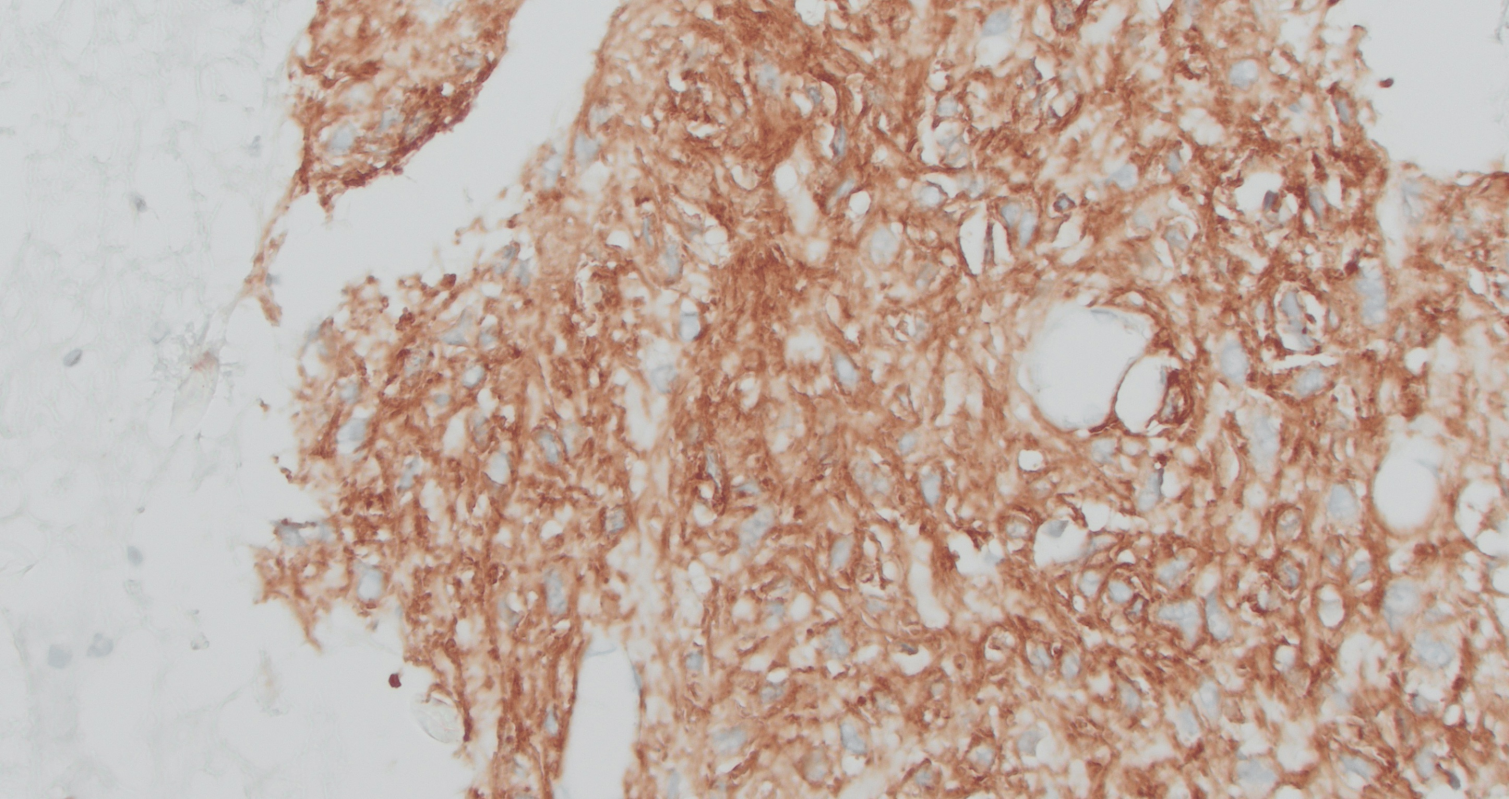
IHC on cell block-Positive for DOG1 (x20)

Immunohistochemistry was positive for DOG-1 (Figure 2) and CD34 but negative for CD117 (c-KIT), desmin, SMA, S-100, GATA-3, ALK-1, and β-catenin. Because of the negative CD117 staining result, further molecular testing was conducted. Fluorescence in situ hybridization analysis revealed no MET amplification or PTEN deletion. Neogenomics analysis revealed a PDGFRA D842V mutation with no RNA fusions or other significant genetic alterations. Immuno-oncology biomarkers revealed microsatellite stability, absence of PD-L1 expression, and a low tumor mutation burden. No BRAF, HRAS, KIT, KRAS, NF1, NRAS, or SDHB mutations were found.

**Figure 3:**
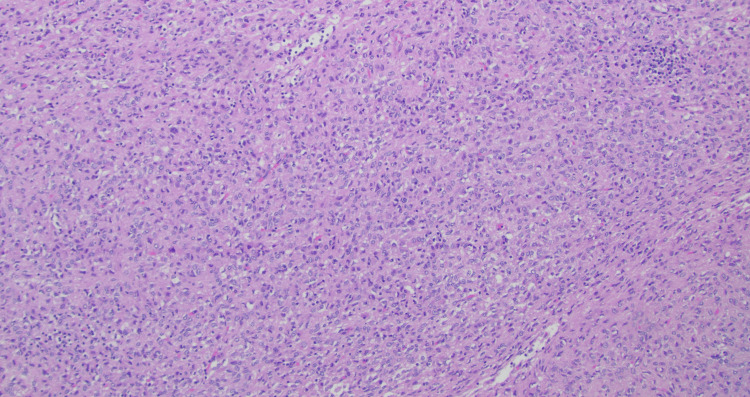
Neoplasm showing spindle cells arranged in fascicles and nests, Cells show moderate to high nuclear pleomorphism (H&E x 10).

The patient was presented at our hospital’s multidisciplinary board and scheduled for GIST excision before initiating radiation therapy for her breast. Gross examination revealed an 8.5 × 7.5 × 4.5 cm encapsulated mass with an attached bowel segment and lobulated yellow omental fat. The dissected surface was tan, fleshy, and cerebriform, with no gross necrosis. Histologic examination confirmed a neoplasm with spindle cells arranged in fascicles and nests (Figure 3).

**Figure 4:**
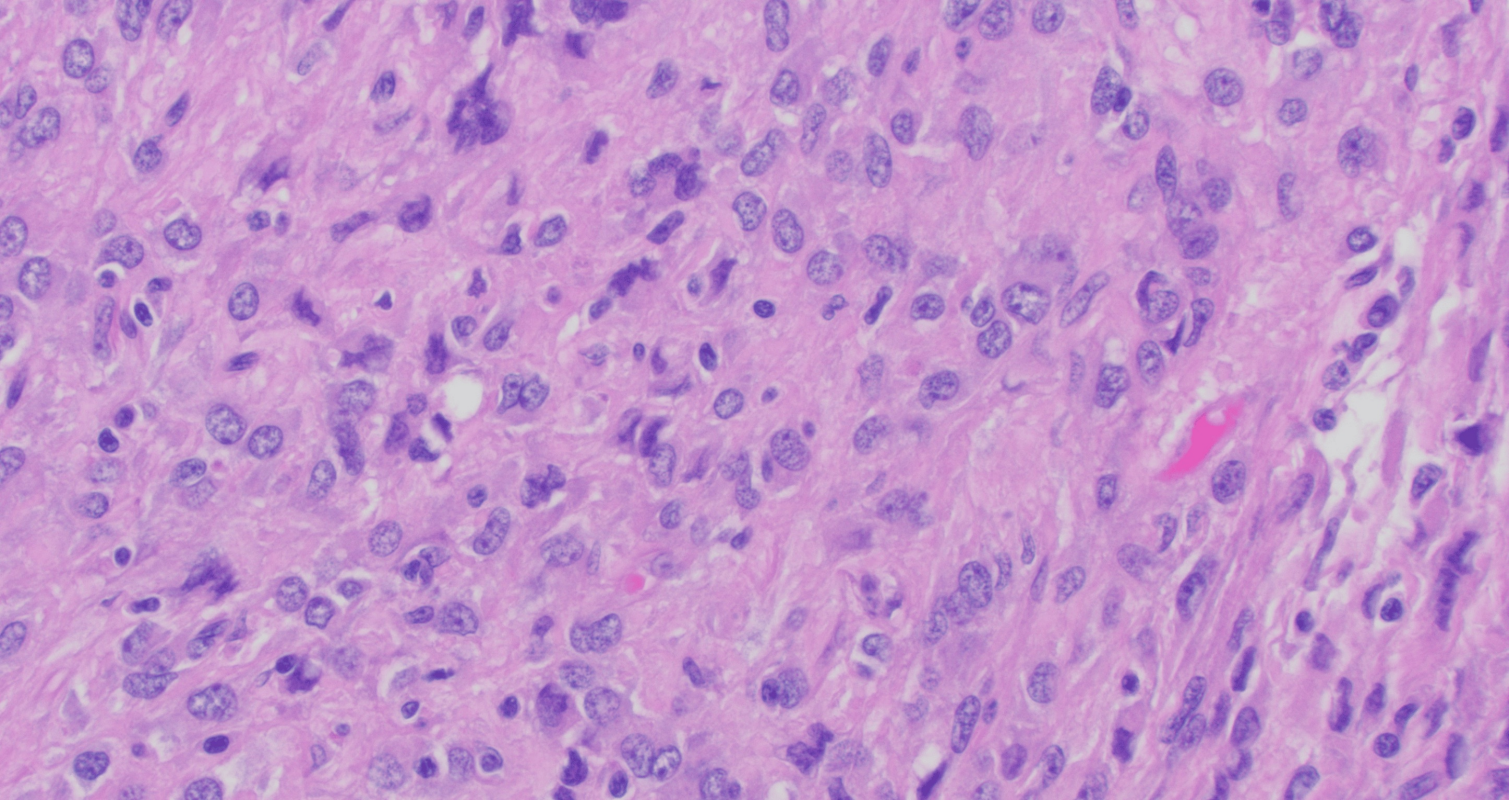
Neoplasm showing spindle cells arranged in fascicles and nests, Cells show moderate to high nuclear pleomorphism (H&E x 40).

**Figure 5:**
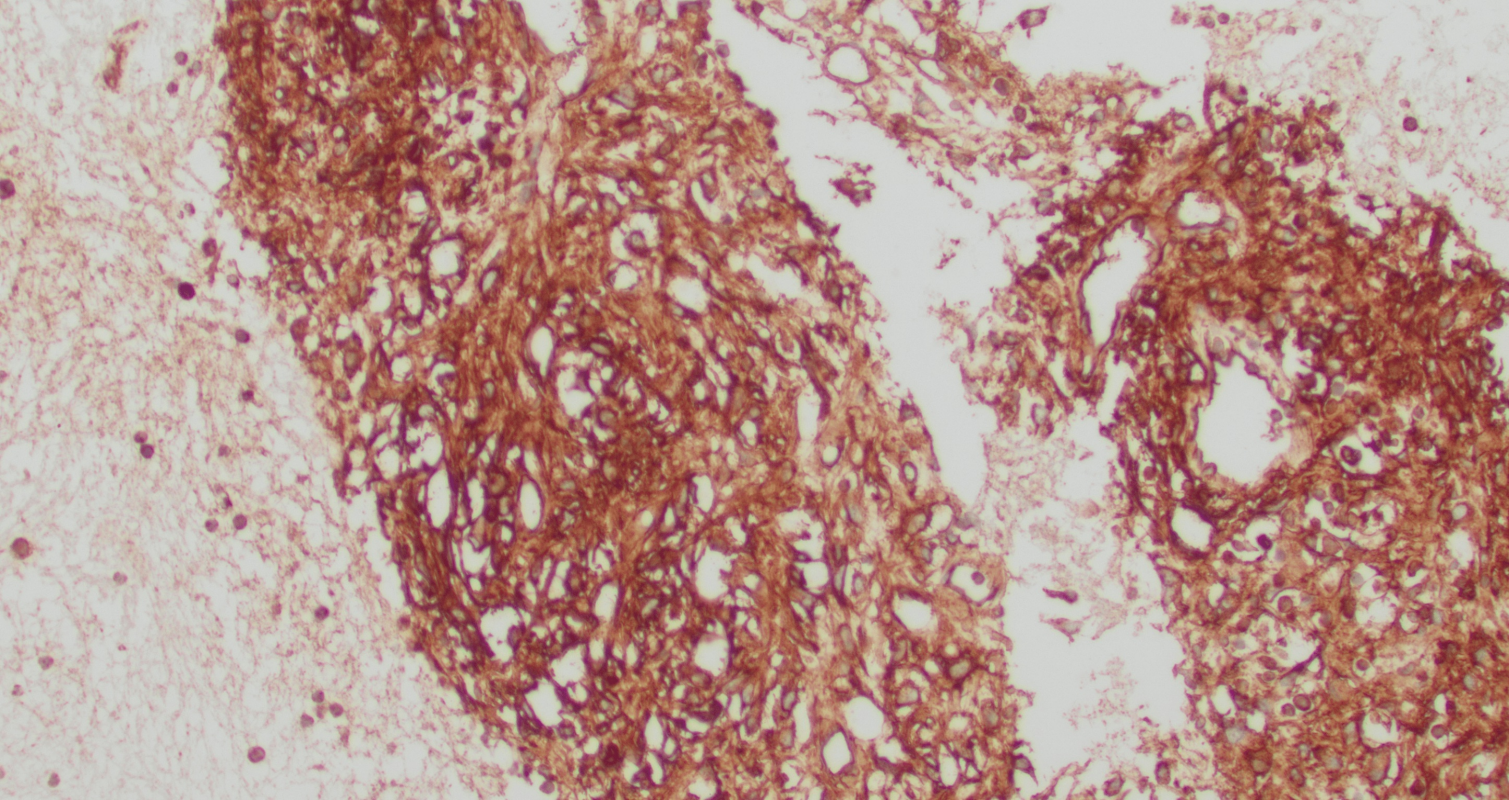
Neoplasm showing positivity for CD34 (x20)

**Figure 6:**
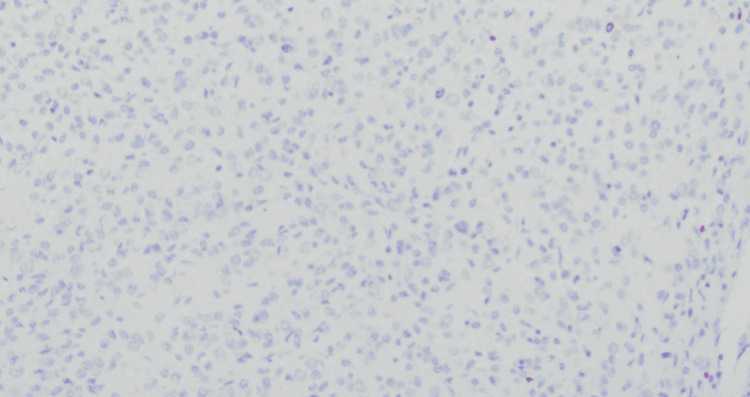
Neoplasm showing less than 5% mitotic index (x10)

However, several foci with moderate nuclear pleomorphism were seen (Figure 4). Mitotic cells were fewer than two cells per 5 mm², and all surgical margins were negative for GIST. No regional lymph nodes were identified. The neoplastic cells were positive for DOG-1 and CD34 (Figure 5). The Ki-67 index was less than 5% (Figure 6).

A second opinion from Brigham and Women’s Hospital in Boston was sought, mainly to rule out a dedifferentiated liposarcoma. Their investigation, which included additional SDHB immunostaining, concluded that pleomorphism in GIST had no prognostic significance beyond the standard risk stratification criteria. Thus, the tumor was classified as low risk for metastasis based on tumor site, size, and low mitotic rate. The final diagnosis was low-grade GIST, with a pathologic stage of pT3 N n/a M n/a.

## Discussion

The recognition of GISTs in the 1990s inspired significant advancements in understanding their origins, tyrosine kinase mutations, and responses to targeted therapy [3]. GISTs, which are common mesenchymal tumors of the gastrointestinal tract, are believed to originate from precursor cells of the interstitial cells of Cajal [1]. They typically appear as well-defined, subserosal, or submucosal masses. Histologically, GISTs exhibit a spindled morphology in 70% of cases, epithelioid morphology in 20%, and mixed morphology in 10% [3]. GISTs frequently express KIT (95% of cases), DOG-1 (98%), PDGFRA (80%), and CD34 (70%-80%). Expression of SDHA and SDHB is generally preserved, except in SDH-deficient GISTs [4].

The discovery of KIT gain-of-function mutations in 1998 was pivotal, facilitating the development of targeted therapies, with imatinib becoming the first-line treatment for advanced GISTs [4]. Approximately 30% of GISTs are malignant; thus, accurate prediction of malignant potential is crucial for managing the risk of local recurrence or metastasis. Risk stratification, which was first developed by Schaefer et al. [3] and later refined by Miettinen and Lasota [1], involved predicting progression based on tumor size, mitotic rate, and site. However, this stratification is less useful for SDH-deficient GISTs, which often require alternative therapeutic approaches because of their resistance to conventional KIT inhibitors [3].

Pleomorphism in GISTs is rare; however, it can be associated with sarcomatous features, such as nuclear enlargement, hyperchromasia, and high mitotic rates [1]. Numerous studies have highlighted that nuclear pleomorphism, when combined with factors such as mitotic count, necrosis, tumor size, and cellularity, is considered a key prognostic indicator in the risk stratification of GISTs [5,6]. Furthermore, some investigations have demonstrated the utility of nuclear pleomorphism as an independent prognostic factor [2]. Pleomorphism may also occur with dedifferentiation, which is often indicated by the loss of KIT and DOG1 expression, either before or after targeted therapy [7]. However, Isidro and Hornick [2] demonstrated that pleomorphism in GISTs, even without dedifferentiation, does not independently affect prognosis beyond standard risk stratification.

Influence of SDH deficiency, KIT/PDGFRA mutations, and Ki-67 on chromatin structure, NE dynamics, and tumor proliferation in GISTs

GISTs can show prominent nuclear pleomorphism due to significant irregular nuclear chromatin distribution and structure. Structural integrity of the nuclear envelope, with further complex molecular pathway interactions between succinate dehydrogenase (SDH) deficiency [8], gain-of-function mutations in PDGFRA or KIT can play a prominent role in chromatin shaping. However, these pathways mostly influence nuclear pleomorphism through chromatin remodeling and may not directly influence tumor proliferative index. The latter is more reflected by Ki-67 [9].

SDH Deficiency and Epigenetic Alterations

SDH-deficient GISTs lead to the accumulation of succinate, an oncometabolite that inhibits enzymes involved in DNA demethylation. This causes widespread hypermethylation of DNA, leading to condensation of chromatin into tightly packed heterochromatin [8]. This abnormal chromatin structure disrupts lamins, which are crucial for anchoring chromatin to the nuclear periphery. Aberrant dysregulation of lamins causes nuclear pleomorphism with nuclear hyperchromasia, chromatin clumping, and irregular nuclear contours. This can further aggravate genomic instability and tumor progression [10].

KIT/PDGFRA Mutations and Signal Transduction Pathways

Gain-of-function mutations in the KIT or PDGFRA genes can cause constitutive activation of many signaling pathways, which include MAPK, PI3K-AKT-MTOR, and STAT3 pathways [8]. These pathways drive cellular proliferation, survival, and differentiation by modulating lamin expression, nuclear envelope transmembrane (NET) proteins, including emerin and MAN1, as well as the LINC complex components like SUN proteins and nesprins. Aberrant signaling can alter lamin A/C levels, affecting the structural integrity of the nuclear envelope and disrupting chromatin organization. These pathways also influence the function of NETs, thereby altering signal transduction, chromatin positioning, and chromatin remodelling [8,10].

Ki-67 and Tumor Proliferative Index

Nuclear pleomorphism and chromatin irregularities, though indicative of malignancy, do not necessarily correlate with the tumor’s proliferative capacity [9,10]. Direct marker of cell proliferation is indicated by Ki-67, where the nuclear protein is expressed during all active phases of the cell cycle (G1, S, G2, and M phases). Ki-67 expression is regulated by key cell cycle pathways, including MAPK/ERK, PI3K-AKT, and E2F. High Ki-67 levels correlate with increased tumor aggressiveness and mitotic potential, making it a more important marker for assessing the proliferative index of GISTs [9].

Hypothesis

Nuclear Pleomorphism and Proliferation in GISTs - Independent Pathways for Chromatin Remodeling and Proliferation

The hypothesis posits that molecular pathways culminating in chromatin remodeling and proliferation in GISTs are driven by SDH deficiency and PDGFRA/ KIT mutations. They are partly independent of the tumor’s proliferative index as indicated by Ki-67. While remodeling of chromatin is a reflection of underlying epigenetic and consequent structural changes, Ki-67 marks tumor proliferative activity, driven by cell cycle regulation.

Clinical implications

Nuclear pleomorphism is more apparent during the S phase of a cell cycle; however, severe pleomorphism may serve nothing more than a plain histomorphologic correlate of chromatin disruption and disorganization. Ki-67 is a cell cycle marker with a more direct prognostic index reflective of all phases (G1, S, G2) of the cell cycle, guiding therapeutic decisions. One can also extrapolate this concept of tumor biology to other mesenchymal tumors such as leiomyomas. In the context of STUMPs (smooth muscle tumors of uncertain malignant potential) or bizarre leiomyomas, nuclear pleomorphism does not necessarily indicate the tumor's mitotic potential. However, other factors, such as atypical mitoses and nuclear hyperchromasia, can suggest tumor aggression and have significant therapeutic implications.

## Conclusions

In GISTs, chromatin irregularities can be caused by alterations in lamins, nuclear envelope integrity, and epigenetic re-modelling, which in turn are driven by SDH deficiency and KIT/PDFGRA mutations. Cell-cycle-related proliferative activity measured by Ki-67 is independent of the grade of nuclear pleomorphism. In order to refine clinical decision-making and understand tumor biology, an independent evaluation of proliferative indices and chromatin alterations is necessary. In this case, the tumor had a greatest dimension of 8.5 cm, showed marked nuclear pleomorphism, but had a low mitotic index. Hence, it was classified as a Grade 1 tumor. 

In the present case, the tumor measured 8.5 × 7.5 × 4.5 cm and demonstrated striking nuclear pleomorphism but retained a low mitotic index, leading to a Grade 1 classification. Expert review further confirmed that nuclear pleomorphism alone does not warrant a concern beyond existing risk assessment parameters. Hence, this case reiterates that treatment decisions in GISTs cannot be driven by nuclear pleomorphism as a risk factor. Instead, reliable prognostic assessment should continue to prioritize size, location, mitotic activity, and molecular alterations rather than pleomorphism in isolation.
